# Effects of the concomitant administration of xanthine oxidase inhibitors with zofenopril or other ACE-inhibitors in post-myocardial infarction patients: a meta-analysis of individual data of four randomized, double-blind, prospective studies

**DOI:** 10.1186/s12872-018-0800-x

**Published:** 2018-06-05

**Authors:** Claudio Borghi, Stefano Omboni, Giorgio Reggiardo, Stefano Bacchelli, Daniela Degli Esposti, Ettore Ambrosioni, on behalf of the SMILE Working Project

**Affiliations:** 1 Unit of Internal Medicine, Policlinico S. Orsola, University of Bologna, Bologna, Italy; 2 Clinical Research Unit, Italian Institute of Telemedicine, Varese, Italy; 3 Mediservice S.r.l., Agrate Brianza, Italy; 4 Divisione di Medicina Interna, Policlinico S.Orsola, Via Massarenti 9, 40138 Bologna, Italy

**Keywords:** Acute myocardial infarction, Hyperuricemia, Angiotensin-converting enzyme inhibitors, Xanthine oxidase inhibitors

## Abstract

**Background:**

Oxidative stress is increased in hyperuricemic patients with acute myocardial infarction (AMI). Use of *sulfhydryl* ACE-inhibitors (ACEIs), such as zofenopril or captopril, plus xanthine oxidase inhibitors (XOIs), may potentially result in enhanced antioxidant effects and improved survival.

**Objective:**

We verified the benefit of such combination in a randomly stratified sample of 525 of the 3630 post-AMI patients of the four randomized prospective SMILE (Survival of Myocardial Infarction Long-term Evaluation) studies.

**Methods:**

One hundred sixty-five (31.4%) patients were treated with XOIs (79 under zofenopril, 86 placebo, lisinopril or ramipril), whereas 360 were not (192 zofenopril, 168 placebo or other ACEIs). In these four groups, we separately estimated the 1-year combined risk of major cardiovascular events (MACE, death or hospitalization for cardiovascular causes).

**Results:**

MACE occurred in 10.1% of patients receiving zofenopril + XOIs, in 18.6% receiving placebo or other ACEIs + XOIs, in 13.5% receiving zofenopril without XOIs and in 22.0% receiving placebo or other ACEIs, but no XOIs (*p* = 0.034 across groups). Rate of survival free from MACE was significantly larger under treatment with zofenopril + XOIs than with other ACEIs with no XOIs [hazard ratio: 2.29 (1.06–4.91), *p* = 0.034]. A non-significant trend for superiority of zofenopril + XOIs combination was observed vs. zofenopril alone [1.19 (0.54–2.64), *p* = 0.669] or vs. placebo or other ACEIs + XOIs [1.82 (0.78–4.26), *p* = 0.169].

**Conclusions:**

Our retrospective analysis suggests an improved survival free from MACE in post-AMI patients treated with a combination of an urate lowering drug with antioxidant activity and an ACEI, with best effects observed with zofenopril.

## Background

Reduced myocardial antioxidant activity and increased oxidant damage have been described in animal models of coronary artery disease and heart failure (HF), and markers of oxidative stress are elevated in the same patients [1]. Among potential stimuli that contribute to myocardial failure progression, the over-activation of xanthine oxidase (XO) plays a central role. XO is an enzyme involved in the last two steps of purine catabolism that ultimately results in uric acid production [2]. The degree of activation of XO is directly related to the the circulating levels of uric acid that can be considered as an indirect marker of oxidative stress in several clinical conditions [2]. Serum uric acid is a potent antioxidant, but it can also promote oxidative stress at high concentrations and in surroundings with low pH or low levels of other antioxidants [3]. In vitro, uric acid may affect vascular smooth muscle and mononuclear cells that are important in pathophysiology of cardiovascular (CV) diseases [4]. Indeed, significantly increased serum uric acid levels have been identified in 25% of patients with heart failure and reduced ejection fraction and have been associated with worsening symptoms and increased mortality [5, 6]. Vascular activity of XO may also increase the oxidative stress that underlies endothelial dysfunction. XO inhibition with allopurinol 300 mg once daily for one month was compared with placebo to investigate its potential in endothelial function improvement after HF. Allopurinol significantly ameliorated endothelium-dependent vasodilation and reduced markers of oxidative stress [7]. In patients with HF allopurinol administration improved left ventricular ejection fraction [8], diastolic function, and coronary flow reserve [9] on the short-term, but showed no definitive benefits on the long-term [10]. In a placebo controlled cross-over study involving patients with chronic stable angina, treatment with allopurinol (600 mg per day) for 6 weeks showed anti-ischemic properties [11]. Thus, current evidence suggest that XOIs may have important cardioprotective activities [12].

Zofenopril is a potent sulfhydryl angiotensin-converting enzyme (ACE) inhibitor, characterized by high lipophilicity, selective cardiac ACE-inhibition, and antioxidant and tissue protective activities [13]. The effects of zofenopril treatment in terms of circulating adhesion molecules, oxidative stress parameters and endothelium-dependent vasodilation were analyzed in essential mildly hypertensive patients. Zofenopril has demonstrated a sustained antioxidant activity and reduced endothelial activation compared to carboxylic ACE-inhibitor ramipril and the beta-adrenoceptor blocker atenolol [14]. The analysis of the overall population of more than 3600 patients with coronary heart disease (CHD) and enrolled in the Survival of Myocardial Infarction Long-Term Evaluation (SMILE) project, showed that the treatment with zofenopril was associated with a lower risk of mortality and morbidity as compared to placebo or other ACE-inhibitors. The beneficial effect of zofenopril was associated with some degree of anti-ischemic effects and confirmed in patients with preserved or impaired left ventricular function [15–18].

Considering the importance of the over-activation of XO in presence of hyperuricemia, we investigated the effects of the concomitant andministration of XO inhibitors and ACE-inhibitors in post-acute myocardial infarction (AMI) patients. We also postulated that the antioxidant activity of zofenopril may be more beneficial in comparison to other ACE-inhibitors in patients with AMI treated with XO inhibitor (XOI). Therefore, in this meta-analysis we assessed subgroups of patients involved in the SMILE studies who were concomitantly treated with XOIs to verify the potential enhanced antioxidant effect and survival improvement after zofenopril treatment.

## Methods

### Study design

The present meta-analysis aimed to evaluate the 1-year combined occurrence of death or hospitalization for CV causes in patients involved in the SMILE studies who were treated with XOIs. Briefly, the SMILE studies were double-blind, randomized, parallel-group studies that compared the efficacy and safety of zofenopril with placebo plus standard therapy for AMI excluding ACE-inhibitors (SMILE-1 and 3) [15, 18], lisinopril (SMILE-2) [17] or ramipril (SMILE-4) [16]. The inclusion criteria for the SMILE studies were as follows: i) early AMI (< 24 h), not eligible for thrombolytic therapy because of late admission to the intensive care unit or with contraindication to systemic fibrinolysis (SMILE-1) [15], ii) confirmed diagnosis of AMI and a prior thrombolytic treatment within 12 h of the onset of clinical symptoms of AMI (SMILE-2) [17]; iii) recent AMI (within 6 ± 1 weeks) with preserved left ventricular ejection fraction (> 40%), treated with a thrombolytic treatment and with ACE-inhibitors (SMILE-3) [18]; and iv) early AMI (< 24 h), treated or not with thrombolysis, with primary percutaneous transluminal angioplasty or coronary artery by-pass graft, and with clinical and/or echocardiographic evidence of left ventricular dysfunction (SMILE-4) [16]. The main exclusion criteria were: i) the presence of cardiogenic shock (Killip class IV); ii) severe hypotension (SBP < 90–100 mmHg), iii) severe hypertension (DBP > 115 mmHg and/or SBP > 200 mmHg) iv) a serum creatinine concentration > 2.5 mg/dL or renal failure; v) bilateral renal artery stenosis; vi) hemodynamically significant valvular disease; vii) current treatment with ACE-inhibitors or angiotensin II-receptor blockers (or acetylsalicylic acid for the SMILE-4), and hypersensitivity to these drugs; viii) inability or unwillingness to give informed consent.

Patients were studied between January 1991 (SMILE-1 study) and July 2009 (SMILE-4 study) and randomized double-blind to receive zofenopril, placebo, lisinopril or ramipril, in addition to standard recommended therapy for AMI. Duration of treatment and follow-up were depending on the study and ranged 6 to 48 weeks.

Patients who at the randomization visit were treated with preparation inhibiting uric acid production, namely allopurinol, tisopurine, febuxostat and other anti-gout medications (collectively defined in this paper as XOIs) were allowed to continue this therapy during the studies.

All studies were conducted according with the Guidelines for Good Clinical Practice and the Declaration of Helsinki and were approved by the Ethics Committee of each participating center. Written informed consent was obtained from each patient before enrollment.

### Statistical analysis

The primary study endpoint was the 1-year combined occurrence of death or hospitalization for CV causes (major CV events or MACE) and it was calculated after weighing for the number of subjects contributing from each study. All patients treated with at least one dose of study medication and providing at least once the measure of the primary efficacy assessment were included in the analysis. The efficacy endpoint was compared across four different treatment groups: patients taking zofenopril with concomitant XOIs, zofenopril without XOIs, placebo or any other ACE-inhibitor (lisinopril or ramipril) plus XOIs, placebo or other ACEI-inhibitors without XOIs. Since in the pooled dataset of the SMILE Studies the number of subjects treated with XOIs was small (165, of which 79 treated with zofenopril and 86 with placebo, lisinopril or ramipril) compared to those not treated with XOIs (3465, of which 1729 under zofenopril and 1736 under placebo or other ACE-inhibitors), the control group was re-sampled with a 2:1 ratio, using a stratified random sampling method, in order to allow a balanced comparison across the four study groups.

Hazard ratios (HRs) and 95% confidence intervals (CIs) were calculated by a Cox proportional-hazard regression model in which treatment group, gender (males vs. females), age and concomitant cardiovascular risk factors (previous angina, congestive heart failure, arterial hypertension, diabetes mellitus, hypercholesterolemia, peripheral arterial occlusive disease, coronary revascularization) were included as a covariate. In order to account for different duration of follow up among the four studies, the relative risk of CV morbidity and mortality was assessed using a time-dependent Cox regression model and corresponding survival curves were drawn. In addition, a confirmatory Kaplan-Meier survival analysis with Log Rank (Mantel-Cox) test was run by considering events at the time of their occurrence, without applying any missing handling procedure.

Furthermore, we ran a confirmatory analysis by using the propensity score approach to balance non-equivalent study groups on observed potential confounding variables, in order to get more accurate estimates of the effect of treatment with XOIs. The propensity score was estimated by applying a logistic regression analysis, where the outcome was the treatment variable and predictor variables were the covariates [19, 20]. Accordingly, we estimated propensity scores with the observed covariates as predictors, and treatment assignment (dummy coded 0 = no treatment with XOIs, 1 = treatment with XOIs) as dependent variable. The selected model included as predictors: age, gender, body mass index, blood pressure, cardiovascular medical history, diagnosis of diabetes, diagnosis of hypertension, fasting plasma glucose, total and HDL cholesterol. After fitting the model according to a stepwise approach, the patients in the two treatment groups were stratified according to the aforementioned predictors and ranked in five equal-sized strata or quintiles (Q). The Q I represented patients with the best and the Q V included those with the worst risk profile; thus, patients inside each Q had a similar overall risk. The choice of five strata was based on the Cochran method [21], which showed that five strata are able to remove approximately 90% of the bias due to a single continuous covariate [22]. To validate the propensity score model we tested each of the covariates (predictors) in a two way (2 conditions × 5 strata) analysis of variance (continuous variables) or using a logistic regression (dichotomous categorical variables).

Homogeneity of patients’ baseline characteristics were compared by a Chi-square test (discrete variables) or a Student t-test (continuous variables). The minimum level of statistical significance was *p* <  0.05. Data were represented as mean ± SD or 95% confidence interval or as absolute (n) and relative (%) frequencies.

## Results

### Patient population

**Fig. 1:**
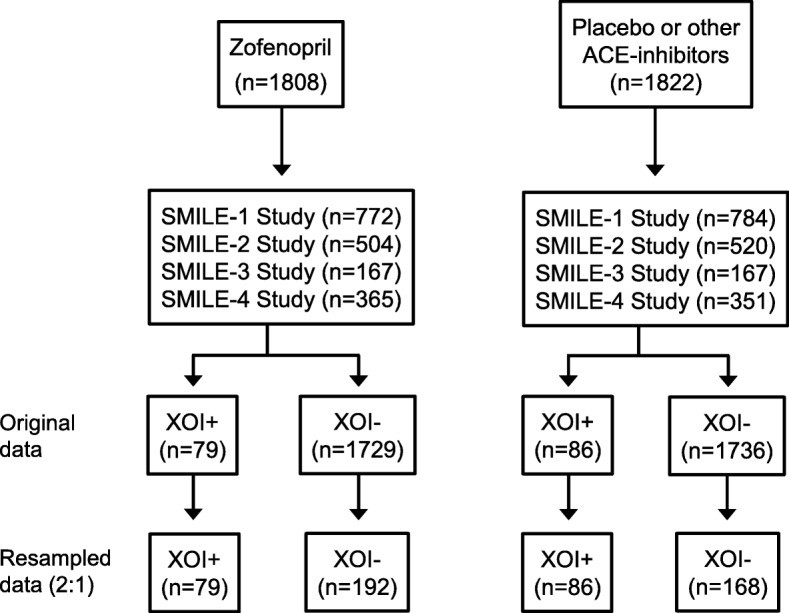
Flow-chart of the patients in the study. ACE: Angiotensin Converting Enzyme; XOI+: patients treated with xanthine oxidase inhibitors; XOI-: patients not treated with xanthine oxidase inhibitors

The pooled data set included in this analysis consisted of 525 patients of whom 271 (51.6%) were treated with zofenopril, 147 (28.0%) with placebo and 107 (20.4%) with ramipril or lisinopril. One-hundred-sixty-five (31.4%) of the 525 patients were treated with XOIs (79 under zofenopril and 86 under placebo or other ACE-inhibitors), whereas the remaining 360 (68.6%) were not (192 zofenopril and 168 placebo or other ACE-inhibitors) (Fig. 1).

Demographic and clinical baseline characteristics were comparable among the four groups except for the prevalence of diabetes and differences in body mass index and diastolic blood pressure (Table 1). Regarding the drugs used during the study no statistically significant difference was observed in the use of cardiovascular therapy across the four study groups (Table 2).

**Table 1:** Demographic and clinical characteristics of the study population summarized by type of treatment (with, + or without, − concomitant xanthine oxidase inhibitors)

	Zofenopril+ (*n* = 79)	Zofenopril-(*n* = 192)	Placebo or other ACE-inhibitors+ (*n* = 86)	Placebo or other ACE-inhibitors- (*n* = 168)	*p*-value
Age (years)	62.0 ± 10.3	60.2 ± 10.2	62.0 ± 10.3	62.9 ± 11.0	0.112
Gender					
*Male*	*57 (72.2)*	*151 (78.6)*	*71 (82.6)*	*119 (70.8)*	*0.121*
*Female*	*22 (27.8)*	*41 (21.4)*	*15 (17.4)*	*49 (29.2)*	
BMI (kg/m^2^)	26.8 ± 3.3	28.2 ± 4.2	27.4 ± 3.9	26.3 ± 3.8	< 0.001
Systolic BP (mmHg)	139.8 ± 22.4	134.0 ± 20.0	135.5 ± 21.0	136.4 ± 22.8	0.251
Diastolic BP (mmHg)	85.2 ± 13.8	80.8 ± 12.3	83.1 ± 12.3	83.3 ± 11.9	0.046
Heart Rate (bpm)	78.9 ± 14.5	77.7 ± 16.1	77.6 ± 16.1	76.1 ± 15.1	0.503
LVEF (%)	46.6 ± 8.4	44.9 ± 10.0	45.1 ± 5.9	45.1 ± 13.2	0.946
Killip Class					
I	38 (48.1)	80 (41.7)	44 (51.2)	75 (44.6)	0.477
II	35 (44.3)	101 (52.6)	39 (45.3)	85 (50.6)	0.519
III-IV	4 (5.1)	11 (5.7)	3 (3.5)	8 (4.8)	0.884
CV risk factors	68 (86.1)	154 (80.2)	70 (81.4)	137 (81.5)	0.728
Diabetes	21 (26.6)	44 (22.9)	27 (31.4)	70 (41.7)	0.001
Hypercholesterolemia	19 (24.1)	47 (24.5)	17 (19.8)	38 (22.6)	0.848
Hypertension	47 (59.5)	97 (50.5)	46 (53.5)	87 (51.8)	0.592

Data are shown as absolute (n) and relative (%) frequencies for categorical variables and as means (±SD) for continuous variables. *P*-values refer to the statistical significance of the difference across the four treatment groups. BMI: Body Mass Index; *SD* Standard Deviation, *BP* Blood Pressure, *LVEF* Left Ventricular Ejection Fraction,* CV* Cardiovascular

**Table 2:** Concomitant cardiovascular drug treatments during the follow-up period by type of treatment (with, + or without, − concomitant xanthine oxidase inhibitors

	Zofenopril+ (*n* = 79)	Zofenopril-(*n* = 192)	Placebo or other ACE-inhibitors+ (*n* = 86)	Placebo or other ACE-inhibitors- (*n* = 168)	*p*-value
ACE-inhibitors	2 (2.5)	6 (3.1)	2 (2.3)	3 (1.8)	0.879
Angiotensin II receptor blockers	–	2 (1.0)	–	–	0.323
Beta-blockers	37 (46.8)	95 (49.5)	39 (45.4)	85 (50.6)	0.837
Calcium channel blockers	6 (7.6)	19 (9.9)	6 (7.0)	13 (7.7)	0.814
Diuretics	13 (16.5)	40 (20.8)	20 (23.3)	38 (22.6)	0.682
Nitrates	31 (39.2)	96 (50.0)	50 (58.1)	92 (54.8)	0.067
Antiarrhythmic drugs	6 (7.6)	16 (8.3)	5 (5.8)	13 (7.7)	0.910
Antithrombotic agents (including ASA)	70 (88.6)	151 (78.6)	69 (80.2)	141 (83.9)	0.219
Lipid lowering drugs (including statins)	32 (40.5)	61 (31.8)	28 (32.6)	52 (31.0)	0.480
Other cardiovascular drugs	11 (13.9)	31 (16.2)	12 (13.9)	22 (13.1)	0.866

Data are shown as absolute (n) and relative (%) frequencies. *P*-values refer to the statistical significance of the difference across the four treatment groups. *ACE* Angiotensin Converting Enzyme, *ASA* Acetyl Salicylic Acid

### Cardiovascular outcomes according to treatment group

**Fig. 2:**
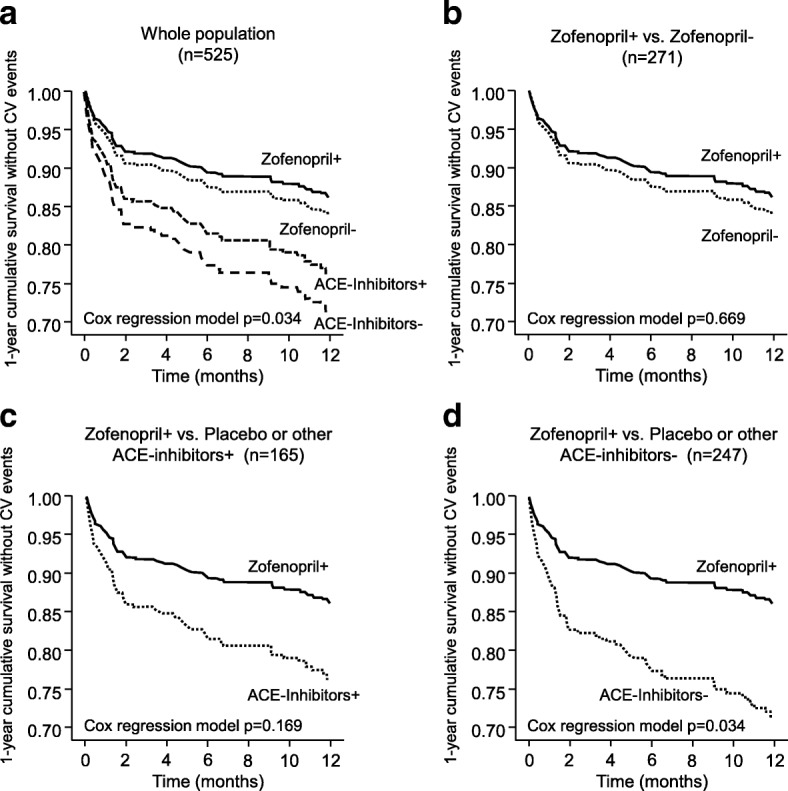
Cumulative survival without events during 1-year of follow-up (based on Cox regression analysis), in (**a**) the whole population of patients treated (+) or not treated (-) with Xantine-oxidase inhibitors, (**b**) patients receiving zofenopril with xanthine oxidase inhibitors (+) as respect to those treated with zofenopril alone (-), (**c**) those treated with zofenopril (zofenopril+) vs. placebo or other angiotensin converting enzyme (ACE)-inhibitors both in combination with and xanthine-oxidase inhibitors (ACE-inhibitors+) and (**d**) those treated with zofenopril and xanthine-oxidase inhibitors (zofenopril+) vs. placebo or other ACE-inhibitors without XOIs (ACE-inhibitors-). *P*-values for the between-group comparison are reported in each panel

MACE occurred in 24 of 165 patients (14.5%) treated with concomitant XOIs and in 63 of 360 patients (17.5%) not treated with XOIs, with a 20% non-statistically significant (*p* = 0.398) lower risk of achieving the end-point under XOIs [hazard ratio: 0.80 (0.48, 1.34)]. Eight (10.1%) patients receiving zofenopril with XOIs, 16 (18.6%) receiving placebo or other ACE-inhibitors with XOIs, 26 (13.5%) receiving zofenopril without XOIs and 37 (22.0%) receiving placebo or other ACE-Inhibitors without XOIs reported a MACE during the study (*p* = 0.034 across groups) (Fig. 2a).

Survival MACE free rate was significantly larger in patients receiving zofenopril with XOIs than in those who were treated with placebo or other ACE-inhibitors without XOIs [hazard ratio: 2.29 (1.06, 4.91), Cox regression analysis *p* = 0.034] (Fig. 2d**)**. A non-significant trend for superiority was observed for zofenopril with XOIs compared to zofenopril alone [1.19 (0.54, 2.64), *p* = 0.669] (Fig. 2b) or to placebo or other ACE-inhibitors with XOIs [1.82 (0.78, 4.26), *p* = 0.169] (Fig. 2c).

**Fig. 3:**
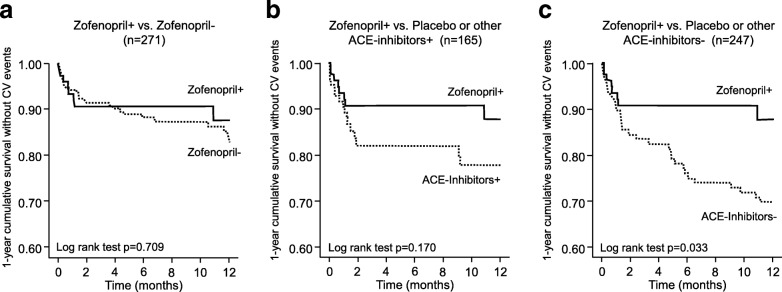
Kaplan-Meier curves for survival without events during 1-year of follow-up, in patients receiving zofenopril with xanthine oxidase inhibitors or XOIs (+) as respect to those treated with zofenopril without (−) XOIs, those treated with placebo or other angiotensin converting enzyme (ACE)-inhibitors plus XOIs, those treated with placebo or other ACE-inhibitors without concomitant XOIs. *P*-values for the between-group comparison based on Log Rank Test are reported in each panel

In the Kaplan-Meier analysis, survival time without any events was significantly longer in patients treated with zofenopril and XOIs [10.9 (10.2, 11.7) months] than in those treated with placebo or other ACE-inhibitors without XOIs [9.5 (8.7, 10.2) months; Log rank test *p* = 0.033) (Fig. 3c). Average survival time free from cardiovascular events was only marginally lower in patients treated with zofenopril without XOIs [10.7 (10.2, 11.2) months; *p* = 0.709 vs. zofenopril plus XOIs) and in those treated with placebo or ACE-Inhibitors with XOIs [9.9 (8.9, 10.8) months; *p* = 0.170 vs. zofenopril with XOIs] (Fig. 3a and b).

### Propensity analysis

A statistically significant difference was observed across the five groups for the predictors included in the propensity analysis, except for body mass index (QI: 27.3 ± 3.6 kg/m^2^; QII: 27.8 ± 3.8 kg/m^2^; QIII: 27.0 ± 3.6 kg/m^2^; QIV: 27.3 ± 4.4 kg/m^2^; QV: 26.7 ± 4.3 kg/m^2^; *p* = 0.329) and the prevalence of diabetes (QI:28.6%; QII: 34.3%; QIII: 37.1%; QIV: 33.3%; QV: 21.0%; *p* = 0.095) and hypercholesterolemia (QI:15.2%; QII: 24.8%; QIII: 25.7%; QIV: 27.6%; QV: 21.9%; *p* = 0.238) (Table 3). After adjusting for the propensity score, the rate of MACE was still non-significantly (*p* = 0.456) lower in XOI-treated patients [hazard ratio: 0.84 (0.34, 2.10)].

**Table 3:** Baseline demographic characteristics of the study population stratified by propensity subgroups (quintiles, Q)

Characteristics	Propensity group					*p*-value for propensity score analysis
	Q I (*n* = 105)	Q II (*n* = 105)	Q III (*n* = 105)	Q IV (*n* = 105)	Q V (*n* = 105)	
	≤0.0095	0.0096–0.0205	0.0206–0.0412	0.0413–0.0849	≥0.0850	
Age (years, mean ± SD)	51.6 ± 8.8	58.1 ± 10.2	61.9 ± 8.2	65.0 ± 7.1	71.4 ± 6.6	< 0.001
Gender (n, %)						
*Male*	*98 (93.3)*	*88 (83.8)*	*87 (82.9)*	*73 (69.5)*	*52 (49.5)*	< 0.001
*Female*	*7 (6.7)*	*17 (16.2)*	*18 (17.1)*	*32 (30.5)*	*53 (50.5)*	
BMI (kg/m^2^, means ± SD)	27.3 ± 3.6	27.8 ± 3.8	27.0 ± 3.6	27.3 ± 4.4	26.7 ± 4.3	0.329
Systolic BP (mmHg, means±SD)	127.4 ± 18.9	129.8 ± 19.0	139.1 ± 20.1	138.5 ± 23.0	144.4 ± 22.2	< 0.001
Diastolic BP (mmHg, means±SD)	77.1 ± 11.7	79.8 ± 11.4	83.3 ± 11.1	84.7 ± 12.6	88.5 ± 12.4	< 0.001
Heart rate (bpm, means ± SD)	73.1 ± 13.1	74.5 ± 13.0	74.1 ± 12.6	81.0 ± 14.8	83.9 ± 15.4	< 0.001
LVEF (%, means ± SD)	48.8 ± 10.2	45.9 ± 10.1	45.7 ± 9.1	42.7 ± 9.4	41.1 ± 10.3	0.002
CV Risk Factors (n, %)	48 (45.7)	80 (76.2)	93 (88.6)	103 (98.1)	105 (100.0)	< 0.001
Diabetes (n, %)	30 (28.6)	36 (34.3)	39 (37.1)	35 (33.3)	22 (21.0)	0.095
Hypercholesterolemia (n, %)	16 (15.2)	26 (24.8)	27 (25.7)	29 (27.6)	23 (21.9)	0.238
Hypertension (n, %)	29 (27.6)	50 (47.6)	61 (58.1)	66 (62.9)	71 (67.6)	< 0.001

Data are shown as absolute (n) and relative frequencies (%) for categorical variables and as means (±SD) for continuous variables. *P*-values refer to the statistical significance of the difference across the five Qs. *SD* Standard Deviation, *BMI* Body Mass Index, *LVEF* Left Ventricular Ejection Fraction, *SBP* Systolic Blood Pressure, *DBP* Diastolic Blood Pressure, *HR* Heart Rate

The rate of MACE significantly (*p* = 0.043) increased at increasing Q. Differences in the effect of the various study drugs were observed within each Q of the propensity score. A superior effect of concomitant treatment with XOIs (and in particular of zofenopril with XOIs) vs. treatment without XOIs (in particular placebo or ACE-inhibitors without XOIs) was observed in Q I (MACE under zofenopril plus XOIs: 0% vs. 20.8% under placebo or other ACE-inhibitors without XOIs) and Q II (4.5% vs. 17.2%) low risk category and in the Q IV (16.7% vs. 28.1%) and Q V (20.0% vs. 25.5%) high risk category.

**Fig. 4:**
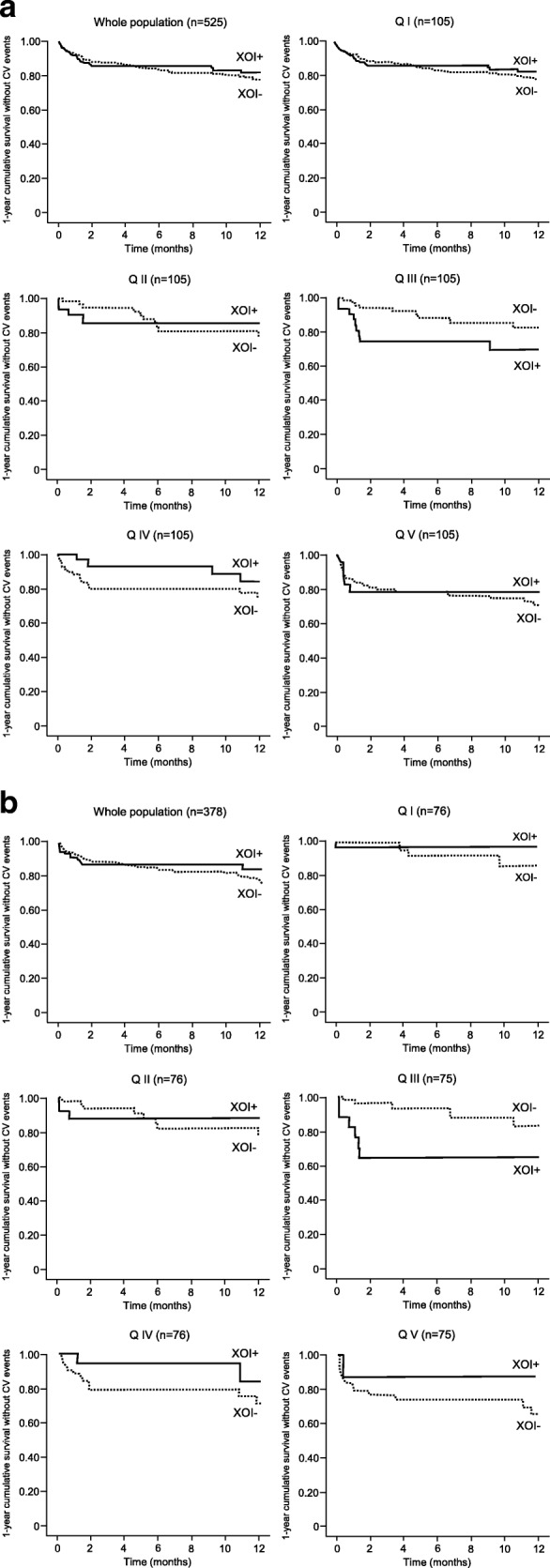
Cumulative survival without events during 1-year of follow-up in the whole study population and after stratification for propensity score (quintiles, Q), according to presence (+) or absence (−) of concomitant treatment with xanthine oxidase inhibitors XOIs. Data are shown for all treated patients (**a**) and after excluding patients treated with placebo (**b**)

Figure 4a, shows cumulative survival without events during 1-year of follow-up in the whole study population and after stratification for propensity score in all the treated patients: the trend for a superior effect of treatment including a XOI was observed for all Qs except for Q III. When patients treated with placebo were excluded from the analysis (thus including only patients treated with zofenopril or other ACE-inhibitors), the analysis confirmed a trend to a higher chance of survival in case of concomitant treatment with XOIs in the lowest and highest Qs, whereas for intermediate risk (QIII) a benefit of XOI treatment was not observed (Fig. 4b).

## Discussion

This retrospective analysis on SMILE pooled patients demonstrated that the combination of an ACE-inhibitor and XOIs may be protective against MACE after AMI compared to treatment with an ACE-inhibitor without XOIs. A combination treatment of zofenopril and XOI seems to promote a statistically significant longer survival time without any events than treatment with other ACEIs not combined with XOIs, whereas a non-statistically significant trend to a large benefit of the XOI + zofenopril combination is observed vs. the combination of another ACE-inhibitor and XOI or vs. zofenopril alone. Possible explanations for our findings, as well as limitations of the present retrospective analysis, will be discussed in the next paragraphs.

In our population, the prevalence of patients who needed preparation inhibiting uric acid production was slightly higher than gout prevalence reported from 2000 to 2005 in European countries (4.5% vs 1.4%) [23]. This may be explained with the observation that there is an independent relationship between hyperuricemia and AMI [24] and patients with gout have an increased risk of AMI [25].

The analysis of pooled data confirmed the results of individual SMILE studies. In each SMILE study, treatment of AMI patients with zofenopril effectively reduced the risk of mortality and hospitalization for cardiovascular causes compared to placebo and other ACE-inhibitors, such as lisinopril and ramipril [26]. The results of pooled SMILE studies supported the notion that there are differences in terms of efficacy between different ACE-inhibitors, and that, in contrast to previous observations, a class effect is unlikely [27]. As a matter of fact, Hansen et al. investigated the risk of mortality and re-infarction after a first episode of AMI and demonstrated that there was none difference in terms of clinical efficacy between ACE inhibitors. However, in this study zofenopril was not included and the only sulfhydryl ACE inhibitor was captopril [27]. Zofenopril demonstrated higher anti-atherosclerotic and antioxidant effects than captopril in the arterial wall of hypercholesterolemic apoE(−/−) mice [28]. In rat models of experimental cardiac ischemia/reperfusion (IR) injury, zofenopril prevented the changes that usually occurred during IR injury and reduced lipid peroxidation, protein oxidation, and nitric oxide levels as well as XO and myeloperoxidase activities and increased the catalase and superoxide dismutase activities [29]. In untreated normocholesterolemic patients with moderate essential hypertension without clinically evident target organ damage, a 12-weeks treatment with zofenopril significantly reduced malondialdehyde levels, plasma NOx and asymmetrical dimethyl-L-arginine concentrations, compared to enalapril that was ineffective [30]. Consistently, the long term treatment with zofenopril in mildly hypertensive patients resulted in a favorable nitric oxide/oxidative stress profile and showed a slower progression of intima- media thickening of the carotid artery beyond lowering arterial pressure [31].

The present retrospective analysis is based on the observation that clinical benefits of early zofenopril treatment in post-AMI phase are related also to its anti-oxidant properties and, therefore, the combination with XOIs may have an enhanced effect. Previous studies have investigated the opportunity of a therapy with oxypurinol 600 mg daily in addition to an ACE-inhibitors or angiotensin receptor blocker and a beta-blocker in patients with moderate-to-severe HF. Oxypurinol significantly reduced serum uric acid by approximately 2 mg/dL, but did not improve the clinical status in unselected patients and when patients were stratified according to uric acid levels patients with elevated uric acid levels (≥ 9.5 mg/dL, *n* = 108) responded favorably to oxypurinol, whereas patients with UA < 9.5 mg/dL exhibited a trend towards worsening [32]. In our study, patients were clustered according to XOI treatment and not to uric acid levels. Therefore, we selected patients who already needed a preparation inhibiting uric acid production and had a therapy. These patients had major benefits in post-AMI phase when they were treated with zofenopril than lisinopril or ramipril. Further larger studies should be recommended to identify which population of hyperuremic patients may achieve major clinical benefits with zofenopril treatment post-AMI.

This study has some main limitations. First of all, it was a retrospective analysis and the number of patients concomitantly treated with ACE inhibitors and XOI in post-AMI phase was limited. This may explain why some comparisons (e.g. those between zofenopril and other ACE-inhibitors) did not achieve statistical significance. We tried to overcome this limitation in two ways: we down-sampled the control population of patients not treated with XOI, thus balancing the comparisons (yet, however, limited to a small sample), and we applied a propensity score analysis [19]. The latter confirmed the superiority of concomitant treatment with XO in terms of prevention of MACE and supported a better efficacy of a combination including zofenopril. However, we acknowledge that, given the retrospective nature of our study, the sample size could have been underpowered to demonstrate the study goal. Second, despite a very similar design of the four SMILE studies, there were some differences in the inclusion criteria, treatment duration, and follow-up. Our analysis included both thrombolyzed and non-thrombolyzed patients and those with and without left ventricular dysfunction; in addition, clinical effect was observed at different time-points. Thus, the patients considered in this study may be representative of those who could be encountered in post-AMI phase in clinical practice. However, in assessments of differences between treatments, variations in baseline characteristics may decrease the sensitivity of such analyses to show interaction.

Finally, we have no information about the exact type of XOI used by patients and the duration of treatment in the patients of these groups and thus we cannot know whether differences in outcomes may be related to differences in the type or duration of treatment. We also do not have information on serum levels of uric acid at baseline and during the follow-up period and thus we do not know the actual effectiveness of XOI treatment.

## Conclusions

Our retrospective analysis on patients involved in the four SMILE studies suggests that concomitant use of zofenopril and urate lowering drugs with antioxidant activity (XOIs) may further improve survival free from MACE in post-AMI patients. Further prospective studies should be recommended to compare the activity of different ACE-inhibitors in hyperuremic patients and establish a treatment schedule to gain major clinical benefits.

## Abbreviations

ACE: Angiotensin-Converting Enzyme
AMI: Acute Myocardial Infarction
CHD: Coronary Heart Disease
CI: Confidence Interval
CV: Cardiovascular
HDL: High Density Lipoprotein
HF: Heart Failure
HR: Hazard Ratio
IR: Ischemia / Reperfusion
MACE: Major Cardiovascular Events
NOx: Nitric Oxide
Q: Quintile
SD: Standard Deviation
SMILE: Survival of Myocardial Infarction Long-Term Evaluation
XO: Xanthine Oxidase
XOI: Xanthine Oxidase Inhibitors
